# Anthropogenic factors are stronger drivers of patterns of endemic plant diversity on Hainan Island of China than natural environmental factors

**DOI:** 10.1371/journal.pone.0257575

**Published:** 2021-09-29

**Authors:** Zhi-Xin Zhu, Mir Muhammad Nizamani, A. J. Harris, Hua-Feng Wang

**Affiliations:** 1 Hainan Key Laboratory for Sustainable Utilization of Tropical Bioresources, College of Tropical Crops, Hainan University, Haikou, China; 2 Key Laboratory of Plant Resource Conservation and Sustainable Utilization, South China Botanical Garden, Chinese Academy of Science, Guangzhou, China; Chinese Academy of Forestry, CHINA

## Abstract

The roles of natural and anthropogenic factors in contributing to the organization of biodiversity at large and small scales have long been challenging to disentangle, and doing so has never been attempted for the island province of Hainan in China based on patterns of taxonomic and phylogenetic diversity. Therefore, in this study, we evaluated the taxonomic and phylogenetic diversity of endemic plants on the island as a function of anthropogenic and natural variables based on non-metric multi-dimensional scaling (NMDS) ordination and generated generalized linear models (GLMs). We found that the highest phylogenetic diversity (1006) and the lowest mean phylogenetic distance (515.5) was in the provincial capital, Haikou. The NMDS analyses indicated that taxonomic diversity was significantly correlated with industrial revenue (*p* = 0.006) and population (*p* = 0.004). Furthermore, most phylogenetic diversity indices were strongly correlated with population and agricultural revenue, while the sampled natural environmental variables were not significantly correlated with plant diversity indices. These findings indicate that anthropogenic factors are the main present-day driving forces of plant diversity in Hainan, though we did detect a significant latitudinal diversity gradient of richness that likely reflects the historical roles of natural environmental factors in the organization of biodiversity on the island. Overall, our results are alarming for biodiversity of the island and indicate that conservation and sustainable use of endemic plant species must be made a critical priority.

## Introduction

In modern times, it has been frequently debated whether natural or anthropogenic variables are the dominant drivers of patterns of biodiversity within a given area. For example, within the urbanized area of Phoenix, Arizona, USA, Hope et al. [1] found that anthropogenic factors drove urban plant diversity; especially that greater economic activity and higher human population was correlated with greater plant diversity. Similar results were found in studies conducted for urban areas of New Zealand [2], Australia [3], and China [4, 5]. On the other hand, anthropogenic climate change has created new challenges for biodiversity conservation because it leads to exacerbated species loss, with increasing numbers of species facing major threats on a large scale [6, 7]. Therefore, whether natural environmental or anthropogenic factors are stronger drivers of plant diversity at various geographic scales remains unclear.

The Province of Hainan is a tropical island of China that comprises an excellent study system for disentangling the roles of the natural environment and human activities on plant diversity. Hainan lies within the Indo-Pacific region, which is known to be as species-rich as the Neotropics and shares little flora in common with Africa or the Americas [8]. Hainan (hereafter, referring to the main island of Hainan, not satellite islands of the province) harbors 397 endemic plant species [9, 10], accounting for 10.5% of the total number of native plants of the island [11]. However, the percentage of endemic species of Hainan is lower than on other tropical islands, such as Taiwan (19.3%), the Philippines (62.8%), and Madagascar (82.4%), mainly because Hainan separated from the mainland relatively recently, is located in close proximity to the mainland, and is smaller than these other islands. Nevertheless, the density of endemic plants in Hainan, at 0.014 species/km^2^, is similar to other islands; e.g., Taiwan (0.030 species/km^2^), and Madagascar (0.015 species/km^2^). While the island harbors great diversity, it is also densely populated by people (267 person/km^2^) and has nine major cities with populations over 200,000. Hainan has a size of about ca. 32,900 km^2^, meaning that humans and the considerable plant diversity of the island occupy a relatively small space together.

Previous studies on the biodiversity of Hainan have had two major limitations. First, simple diversity metrics have been used, but these ignore the important roles of geographic range and phylogeny in the identification of biodiversity priority areas or hotspots. A better understanding of phylogenetic structure could help with plant species conservation [12], such as by identifying unique, previously undelimited plant communities from an evolutionary perspective and, therefore, facilitating assessment of their need for protection and possibilities for sustainable development. Second, there was little research focused on the phenological patterns of endemic plant species of Hainan, despite that phenology can be a major indicator of looming changes in patterns of biodiversity, especially due to modern anthropogenic climate change [13]. Therefore, more work on the biodiversity of Hainan is needed, especially within a phylogenetic framework.

In this study, we analyzed the correlation between the taxonomic and phylogenetic diversity of endemic plant species of Hainan and natural and anthropogenic environmental variables. Our goal was to determine whether natural or anthropogenic environmental factors are stronger drivers of endemic plant species richness and phylogenetic diversity on the island. The results can be used to improve biodiversity conservation strategies, especially under climate change and continued economic development and urbanization of the island. We focused on the following questions: 1) What are the taxonomic and phylogenetic patterns of plant diversity across different cities in Hainan? and 2) Are natural or anthropogenic environmental factors stronger drivers of those patterns?

## Materials and methods

### Sites

**Fig 1:**
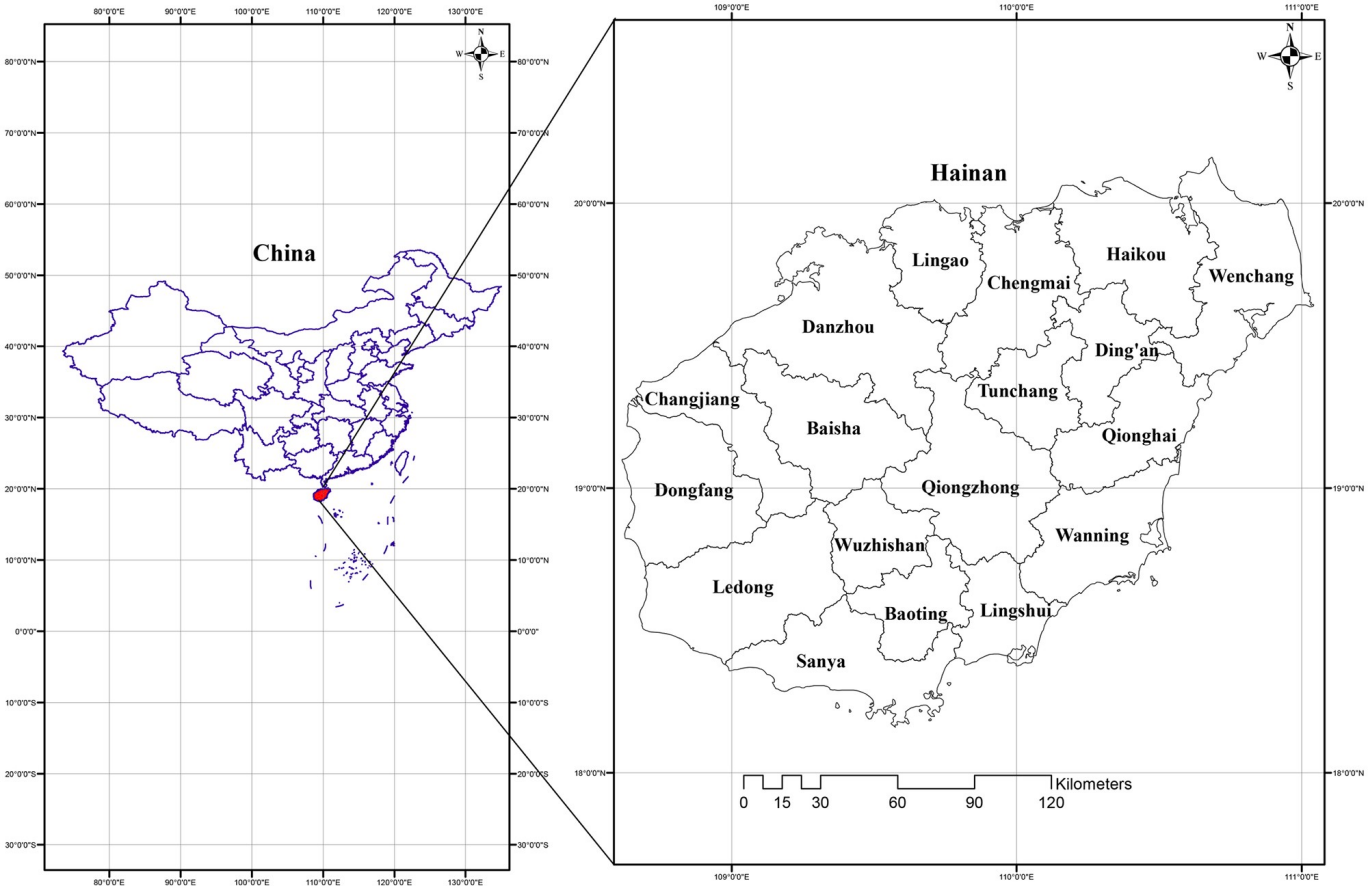
The 18 included administrative units (city or county) of Hainan, China.

Hainan Island is located just south of mainland China, between 19°20’ to 20°4’2" N and 108°9’ to 120°3’ E. It is bordered on the north by the Qiongzhou Strait, which separates it from and Guangdong Province, on the west by the North Bay, and on the south by the South China Sea (Fig 1). Hainan is located on the northern edge of the global tropical zone and has a tropical monsoon climate. Its land area is approximately 35,400 km^2^. On Hainan there are 81 mountains with altitude exceeding 1,000 m above-sea-level^10^.

In this study, we focused only on the main island of Hainan, because we lacked data from its outlying islands, especially comprising four archipelagoes. Within Hainan, there are 20 major urbanized city-level administrative units (i.e., cities or counties) but we do not have data for two, so we only included 18 major urbanized city-level administrative units. However, among these, we lacked plant species and anthropogenic data from Yangpu and Sansha, which are only recently established as urban areas. Therefore, we included 18 administrative units in this study, of which Haikou, Sanya, Danzhou, Wuzhishan, Wenchang, Qionghai, Wanning, and Dongfang are cities, while Ding’an, Tunchang, Chengmai, Lin’gao, Qiongzhong, Baoting, Baisha, Changjiang, Ledong, and Lingshui are counties (Fig 1).

### Data sources

In this study, we initially assessed all 397 endemic plant species of Hainan based on a checklist in Francisco-Ortega et al. [9, 10]. We determined the distribution of each endemic species in each of 18 administrative units within Hainan based on two literature sources, Yang et al. [11] and Xing et al. [14], and specimen records in the Chinese Virtual Herbarium [15]. For some species, we could not confidently determine the distribution (e.g., *Decaspermum teretis* Craven, *Diospyros oliviformis* Miau). Therefore, we omitted these species from the downstream data analyses and included only 387 endemic plant species (bold, S2 Appendix). For these remaining 387, we generated a binary data matrix indicating their presence or absence in each of the administrative units.

We assessed the protection level of each endemic species according to categories assigned by the Ministry of Environment Protection (MEP) and Chinese Academy of Sciences (CAS) [16]. For the rarity or commonality of each species, we used the red list of biodiversity used in China [16] and categorized species as extremely rare, very rare, rare, common, or very common.

### Anthropogenic and natural environmental data

We compiled anthropogenic data from the 2013 Statistical Yearbook of Hainan for each of the administrative units (i.e., 18 counties or cities within Hainan) [17]. We selected a pool of candidate variables including the number of inhabitants (population), agricultural revenue, industrial revenue, retail consumer goods revenue, the number of tourists, savings deposits, and the area (km^2^) at city or county scale resolution within Hainan from 2013 Statistical Yearbook of Hainan [17]. In addition, we used the following natural environmental variables from WorldClim [18]: Max Temperature of Warmest Month (BIO5), Min Temperature of Coldest Month (BIO6), Annual Precipitation (BIO12), and Mean Diurnal Range (BIO2) at each county or city scale (S1 Appendix). We focused on these variables, because they are considered among the most important climatic determinates of plant distributions globally [19–22], while water variables (represented here by BIO12) and diurnal changes (i.e., BIO2) are known to be critical for dynamics of plants of Hainan, specifically [23–25]. The WorldClim values for these variables are averages from 1950 to present using data from weather stations and inferences between stations [26]. We found the averages of the four climatic variables for each county using ArcMap 10.2 [27], in which we determined the geographic boundaries of counties using a non- copyrighted GIS data layer hosted on Data Basin [28] derived from the TIGER/Line and Census TIGER products of the US Census Bureau [29]. We organized the climatic data into two matrices, one each for all counties and counties with floras, where rows represented counties, columns represented the climatic variables, and cells contained the average values of the variables.

### Phylogenetic data

We generated one phylogenetic tree topology based on the 387 endemic plant species of Hainan using the most recent supertree available from Phylomatic [30] of the Phylocom software package [31] We estimated branch lengths for the tree by assigning ages to nodes based on fossil records [32] and then applying the bladj function in Phylomatic. The bladj function generates branch lengths over an ultrametric trees by distributing nodes with unknown ages evenly between nodes with date calibrations. Utilizing branch lengths for phylogenetic diversity metrics is critical because most metrics are highly sensitive to changes in the tree topology when it is used in the absence of branch lengths [33]. We also used “Picante” and “Ape” packages of R3.5.6 to calculate all phylogenetic metrics [34].

Based on the phylogenetic tree, we calculated three phylogenetic metrics among species. These were phylogenetic diversity (PD), mean phylogenetic distance (MPD) and phylogenetic mean nearest taxon distance (MNTD). PD is a sum of branch lengths across the phylogenetic tree. PD is a classic phylogenetic metric, MPD is the average branch length among all pairs of species [35], and MNTD is the average branch length between each taxon and its closest relative on the tree. Thus, PD and MPD are measures of overall phylogenetic structuring of a plant community, while MNTD represents more patterns in more recent diversification.

### Statistical analysis

We determined statistical breaks in the species richness among 18 administrative units using Jenk’s natural breaks [36]. We used an R script to obtain the goodness of fit for two to 18 breaks with the function plotJenks of the GmAMisc library v. 1.1.1 (https://github.com/gianmarcoalberti/GmAMisc) (S3 Appendix). Based on the goodness of fit scores, we selected the least breaks beyond which adding more breaks yielded considerably less gains (i.e., elbow method, S4 Appendix). We also analyzed the latitudinal diversity gradient of endemic species richness by performing linear regression in Microsoft Excel.

For the two datasets of natural and anthropogenic variables, we first determined covariances within each dataset. Based on this analysis, we retained agricultural revenue, geographic area of each administrative unit within Hainan in 2013, the maximum temperature of the warmest month (BIO5), minimum temperature of the coldest month (BIO6), mean annual precipitation (BIO12), and mean diurnal range (BIO2). Thereafter, we conducted non-metric multi-dimensional scaling (NMDS) analyses between the binary data matrix of species presence/absence and the anthropogenic and natural environmental variables. Additionally, we analyzed the relationships between endemic plant species richness and anthropogenic and natural factors of each administrative unit using a multiple general linear model (GLM) with stepwise selection in R [37]. We adopted the GLM the model with the lowest Akaike Information Criterion (AIC).

## Results

### Plant species composition

Based on a prior inventory by Francisco-Ortega et al. [9, 10], there are 397 species of endemic plants on Hainan Island but we analyzed 387 species, for which sequence data were available. These belong to 71 families and 212 genera (S2 Appendix). Among these, the families containing the largest numbers of endemic species are Rubiaceae (33 species), Lauraceae (27), Poaceae (26), Fagaceae (23), Orchidaceae (21), Cyperaceae (15), Euphorbiaceae (14) and Myrtaceae (14), while the genera *Hedyotis* L. (11, Rubiaceae), *Syzygium* P. Browne ex Gaertn. (10, Myrtaceae), *Castanopsis* (D. Don) Spach (eight, Fagaceae), *Cyclobalanopsis* Oerst. (eight, Fagaceae), *Diospyros* L. (eight, Ebenaceae), and *Bambusa* Schreb. (six, Poaceae) had the largest numbers of endemics. We found that 29 species were vines, 89 species were herbs, 122 species were shrubs, 115 species were trees, six species were either herbs or shrubs, and 26 species were shrubs or trees (Table 1). Seven species were annuals, while the other 380 were perennials (Table 2). According to the red list of biodiversity in China [38], two species are extremely rare, 97 species are rare, 203 species are very rare, 59 species are common, nine species are very common, and the frequencies of 17 species are unknown. The list, which was prepared in 2010, includes two globally extinct species (EX; *Lepisanthes unilocularis* Leenhouts and *Begonia sublongipes* Y.M. Shui), which were omitted from analysis, 25 critically endangered (CR) species, such as *Cycas changjiangensis* N. Liu, *Platea parvifolia* Merr. et Chun, 48 endangered (EN) species, such as *Keteleeria hainanensis* Chun et Tsiang, *Reevesia botingensis* H. H. Hsue, and *Syndiclis chinensis* Allen., 43 species vulnerable (VU) species, 51 near threatened (NT) species, 144 species of least concern (LC), and 31 species that are Data Deficient (DD).

**Table 1:** Counts of habits of endemic plants in Hainan.

Categories	No. of species
Vine	29
Herb	89
Shrub	122
Tree	115
Herb/Shrub	6
Shrub/Tree	26
Total	387

**Table 2:** Counts of life histories of endemic plants in Hainan.

Life form	No. of species
Annuals	7
Biennials	0
Perennials	380
Total	387

In Hainan, the flowering and fruiting periods mainly occur in summer and autumn, from April to September, which accounts for 75.97% of the total (Table 3). We found that most species both flower and fruit within the same quarter of the year. Those species with unknown flowering or fruiting periods were primarily bamboos (Poaceae), or Cyperaceae, such as *Fimbristy lischingmaiensis* S. M. Huang.

**Table 3:** Counts and percentages of flowering and fruiting periods of endemic plants in Hainan.

	No. of species	No. of species	Flowering phase percentage (%)	Fruiting period percentage (%)
Jan.-Mar.	34	34	8.786	8.786
Apr.-Jun.	144	48	37.209	12.403
Jul.-Sept.	150	182	38.760	47.028
Oct.-Dec.	57	100	14.729	25.840
Unknown	2	23	0.517	5.943
Total	387	387	100	100

### Distribution of endemic botanical diversity

**Fig 2:**
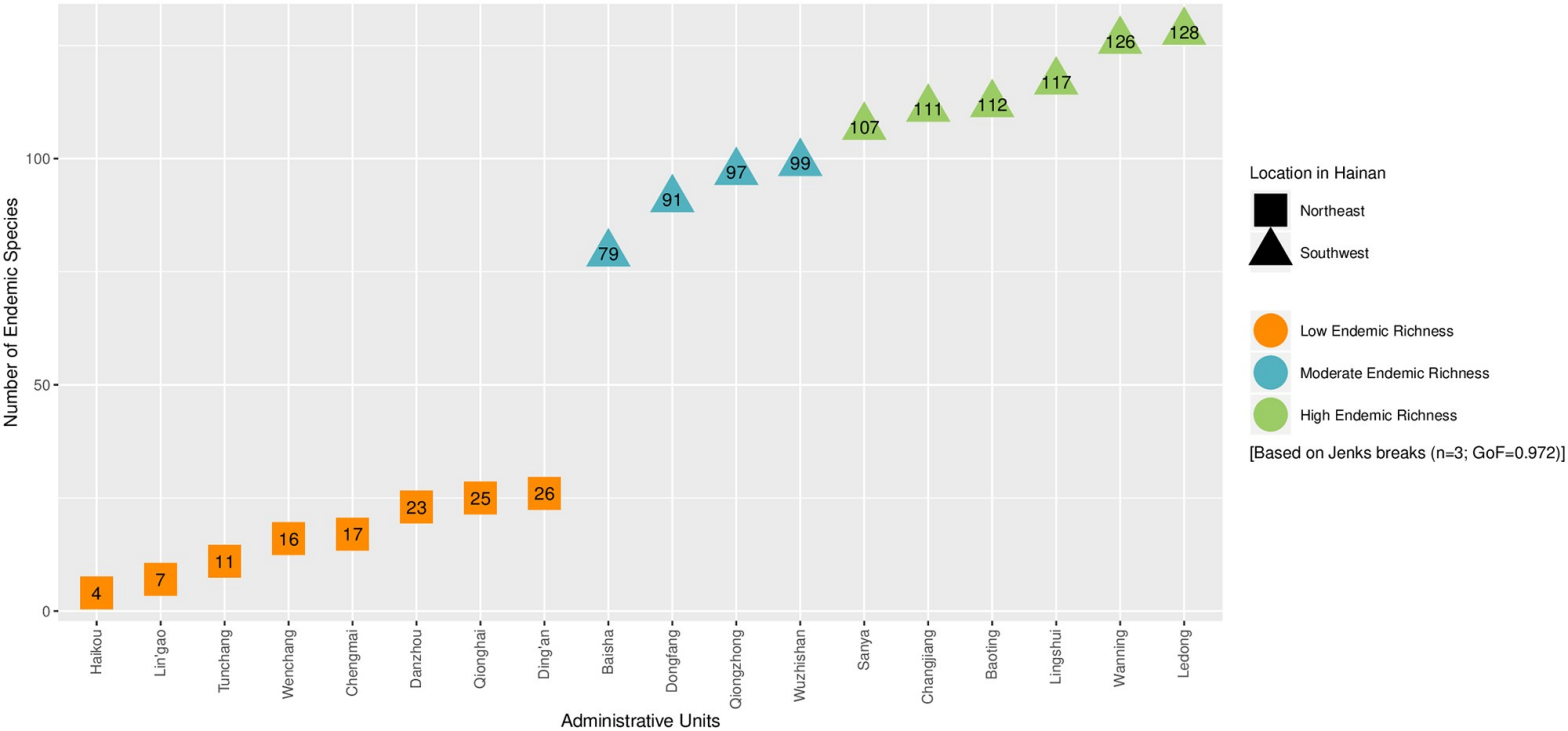
The number of endemic species in each administrative unit (city or county) of Hainan, China.

**Fig 3:**
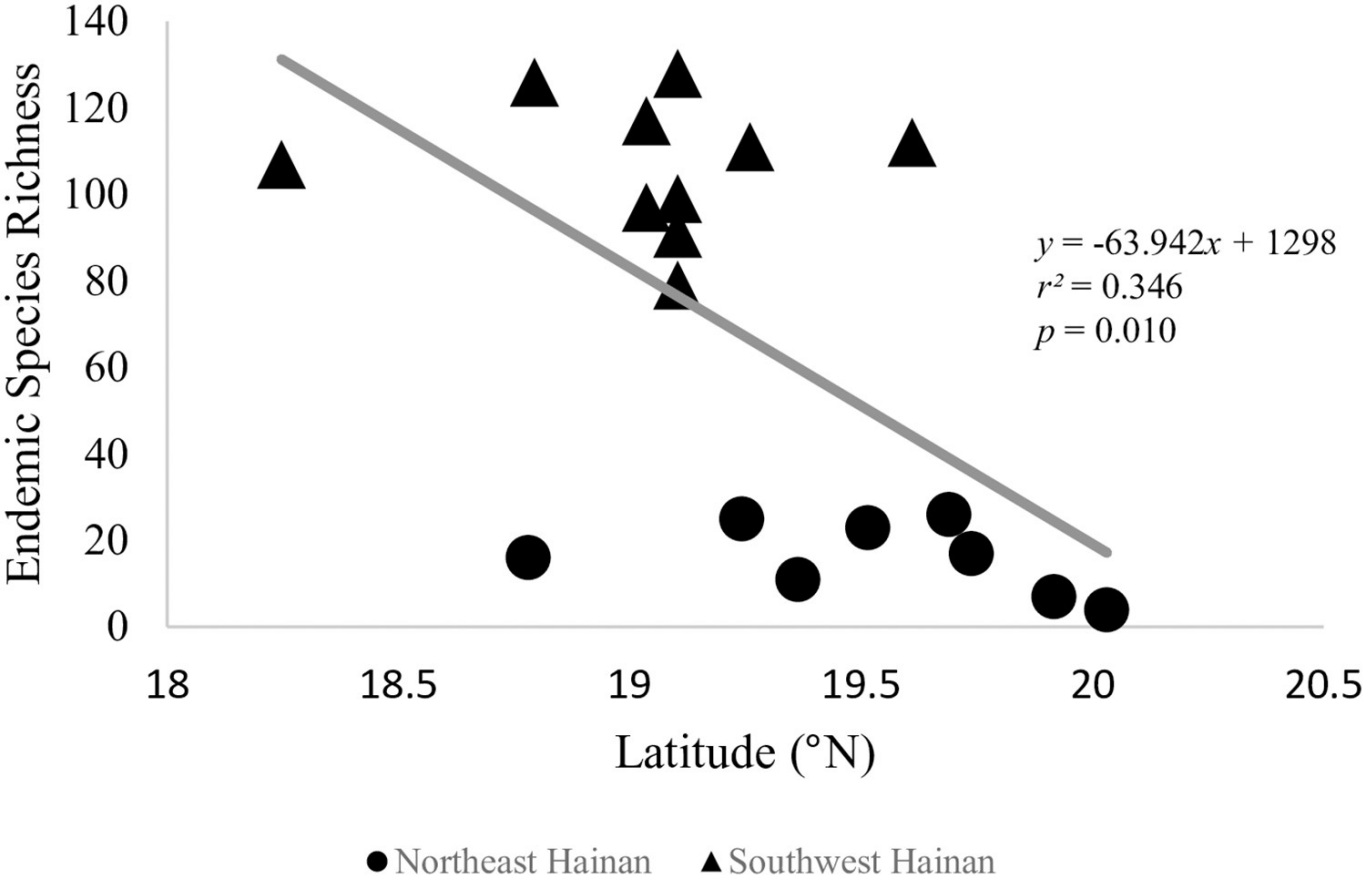
A linear regression analysis revealed that there was a weak but significant negative correlation between species richness and latitude across Hainan (r2 = 0.346, p = 0.010; Fig 3).

Among administrative units, Haikou, which is the capital of Hainan, and Sansha have the fewest species with four and two, respectively, while Wanning and Ledong have the most with 141 and 136 species, respectively (Fig 2). Based on an analysis of Jenk’s breaks, we discovered that the number of endemic species in Hainan’s northeastern administrative units, namely Haikou, Wenchang, Chengmai, Ding’an, Tunchang, Qionghai, Danzhou, and Lin’gao, was significantly lower than in the southwestern units (Fig 2). Among southern units, Jenk’s breaks showed that there were two additional discernable levels of endemic diversity, which we refer to as moderate and high (Fig 2). A linear regression analysis revealed that there was a weak but significant negative correlation between species richness and latitude across Hainan (*r*^*2*^ = 0.346, *p* = 0.010; Fig 3).

We also assessed the distributions of diversity of the endemic species using metrics of phylogenetic diversity. The lowest and highest values for phylogenetic diversity (PD) were for Haikou (1006) and Ledong (8880), respectively. The lowest and highest values of mean pairwise phylogenetic distance between species (MPD) were for Qionghai (257) and Haikou (515.51), respectively. The lowest and highest values of mean nearest taxon distance (MNTD) were in Ledong (110) and Haikou (384), respectively (Table 4).

**Table 4:** The phylogenetic diversity (PD), mean phylogenetic distance (MPD) and mean nearest phylogenetic taxon distance (MNTD) of endemic plants in 18 administrative units of Hainan, China.

Administrative Unit	ntaxa	PD	MPD	NRI	*p*-_MPD_	MNTD	NTI	*p*-_MNTD_
Baisha	79	6865	306.5	-0.7	0.763	139.593	-1.8	0.966
Baoting	112	8697	290.4	1.023	0.158	121.901	-0.7	0.737
Changjiang	111	8716	284.2	1.804	0.037	125.82	-1.4	0.916
Chengmai	17	1947	289	0.412	0.327	162.89	0.6	0.267
Danzhou	23	2593	290	0.413	0.34	174.546	-0.7	0.766
Ding’an	26	2657	298.9	0.03	0.521	144.453	0.81	0.207
Dongfang	91	7696	294.8	0.485	0.33	136.116	-1.9	0.971
Haikou	4	1006	515.5	-3.515	0.986	383.518	-2.7	0.989
Ledong	128	8880	284.3	1.993	0.022	110.095	0.79	0.214
Lin’gao	7	1147	325.4	-0.649	0.817	228.019	-0.4	0.651
Lingshui	117	8773	292.6	0.802	0.225	116.804	0	0.487
Qionghai	25	2478	257.2	2.068	0.013	145.234	0.77	0.223
Qiongzhong	97	7599	292.7	0.69	0.251	119.052	0.32	0.385
Sanya	107	8084	295.9	0.364	0.385	115.956	0.45	0.323
Tunchang	11	1234	295.5	0.099	0.486	124.609	2.35	0.007
Wanning	126	9265	298.3	0.112	0.479	112.203	0.54	0.314
Wenchang	16	1633	284.2	0.568	0.27	135.626	1.82	0.03
Wuzhishan	99	7694	297	0.262	0.401	123.061	-0.4	0.625

Positive values indicate PD was over-dispersed with *p*>0.95, Negative values indicate PD was clustered with *p* < 0.05. We ran 999 randomizations to assess significance. NRI = net relatedness index, NTI = nearest taxon index.

In the analyses of MPD, we found that phylogenetic diversity was significantly over-dispersed in Haikou [net relatedness index (NRI) < 0, *p* > 0.95]. However, in the MNTD analyses, we found that the phylogenetic diversity was significantly over-dispersed in Baisha, Dongfang, and Haikou [nearest taxon index (NTI) < 0, *p* > 0.95)], and was significantly clustered in Tunchang, Wenchang and Sansha (NTI > 0, *p* < 0.05 (Table 4).

### Drivers of plant taxonomic and phylogenetic diversity

**Fig 4:**
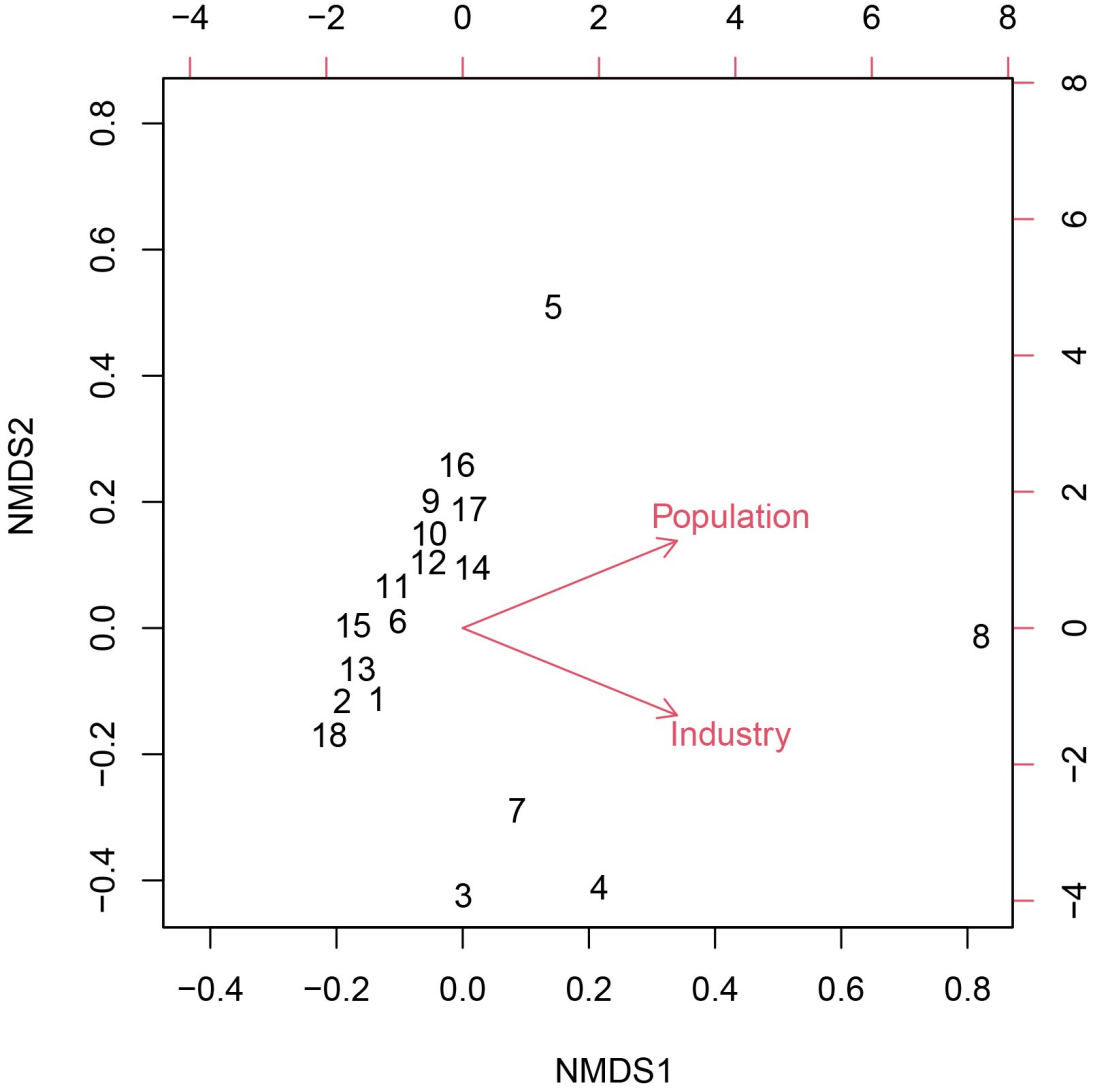
Results of non-metric multidimensional scaling (NMDS) analyses of the endemic species of 18 administrative units and the environmental variables. Industry = industrial revenue.

We determined that plant taxonomic diversity was significantly correlated with the anthropogenic environmental variables industry revenue (*p* = 0.006) and population (*p* = 0.004) (Table 5, Fig 4). However, we found there were no natural environmental variables significantly correlated with plant taxonomic diversity across the 18 administrative units in Hainan Island. Furthermore, most phylogenetic diversity metrics (excluding PD and NTI) were strongly correlated (*r*^*2*^ > 0.8) with population and agricultural revenue, while natural environmental variables were not significantly correlated with these metrics (Table 6).

**Table 5:** Non-metric multidimensional scaling (NMDS) analyses of the endemic species in 18 administrative units and the environmental variables.

	NMDS1	NMDS2	*r* ^2^	*p*
Population	0.37034	-0.9289	0.581	0.004**
Agricultural revenue	0.6946	-0.7194	0.0328	0.772
Industrial revenue	0.13103	-0.99138	0.6103	0.006**
Number of tourists	-0.06874	-0.99763	0.2661	0.132
Area	-0.23208	-0.9727	0.0568	0.606
Min Temperature of Coldest Month (BIO6)	-0.3377	0.94125	0.2007	0.169
Max Temperature of Warmest Month (BIO5)	-0.41259	-0.91092	0.2083	0.191
Annual Precipitation (BIO12)	-0.2701	0.96283	0.1222	0.361
Mean Diurnal Range (BIO2)	-0.98448	-0.17547	0.096	0.482

Significance assessed with 999 permutations. Significance codes: 0 = ’***’, 0.001 = ’**’, and 0.01 = ’*’. As Yangpu and Sansha data were lacking, they were both excluded in the data analyses.

**Table 6:** Results of generalized linear model (GLM) analyses between the phylogenetic diversity index and anthropogenic/natural environment variables.

	SRR	PD	MPD	NRI	MNTD	NTI
Population	NI	NI	NI	NI	NI	NI
Agricultural revenue	NI	NI	NI	*	NI	*
Industrial revenue	NI	*	**	*	***	*
Number of tourists	NI	NI	NI	NI	NI	NI
Area	NS	NI	NI	NI	NI	NI
Min Temperature of Coldest Month (BIO6)	NS	NI	NS	NI	NI	NS
Max Temperature of Warmest Month (BIO5)	NI	*	NI	NI	NI	NI
Annual Precipitation (BIO12)	NS	NI	NI	NI	NI	NI
Mean Diurnal Range (BIO2)	*	NS	.	.	NI	.
t value	-2.467	-2.355	-2.799	-0.01	10.425	1.314
p	0.0283*	0.0336	0.01507	0.992	0.000	0.2116
AIC	183.98	340.62	159.35	53.469	190.07	53.891

SRR = Species Richness Residues, PD = phylogenetic diversity, MPD = mean phylogenetic distance, NRI = net relatedness index, MNTD = mean nearest phylogenetic taxon distance, NTI = nearest taxon index, NI = not included in the model, NS = not significant in the model, AIC = Akaike information criterion. Significance codes: 0 = ’***’, 0.001 = ’**’, and 0.01 = ’*’.

## Discussion

Within Hainan, Haikou is unique for being the capital and having a longer history of human activities on the island including agriculture, deforestation, urbanization, and other landscape alteration. In modern times, Haikou is one of the most rapidly-developing urbanized areas in Hainan, and its good soils, which may have facilitated high biodiversity, have also made the area attractive for urban development and economic expansion. Presently, Haikou is a highly urbanized land area containing only four endemic species, *Cycas hainanensis* C.J. Chen, *Phyllanthus rheophyticus* M. G. Gilbert & P. T. Li, *Crotalaria yaihsienensis* T. C. Chen, and *Lithocarpus elmerrillii* Chun, and exhibiting the lowest PD (1006) among all 18 administrative units. The urban environment of the city likely acts as a strict habitat filter that limits overall species diversity, including the presence of endemics. With respect to phylogenetic diversity, strict habitat filtering also often leads to phylogenetic clustering, or under-dispersion [39], and this is consistent with observations for other native urban floras, such as throughout Germany [40] and China [41]. However, we found that Haikou exhibited phylogenetic over-dispersion of endemic species of Hainan; for example, MPD and MNTD were greatest for Haikou (515.5 and 384, respectively; Table 2) compared to other cities. This could reflect high, recent rates of local extirpation, which is known to cause patterns of phylogenetic overdispersion [42].

**Fig 5:**
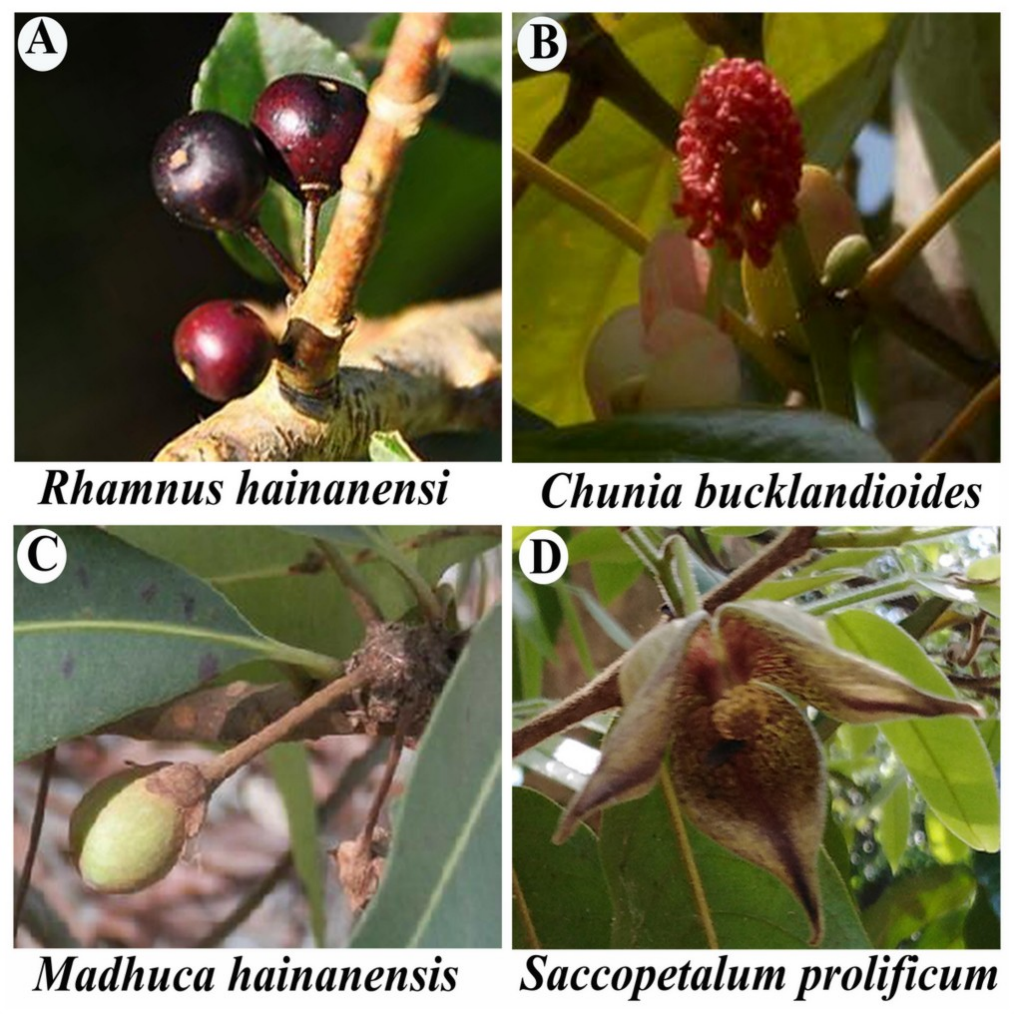
Some representatives of the endemic plant species of Hainan. A, *Rhamnus hainanensi* Merr. et Chun (Rhamnaceae); B, *Chunia bucklandioides* H. T. Chang (Hamamelidaceae); C, *Madhuca hainanensis* Chun et How (Sapotaceae); D, *Saccopetalum prolificum* (Chun & F. C. How) Tsiang (Annonaceae).

In contrast to Haikou, Ledong had the lowest MNTD, which primarily reflects community structing occurring at the tips of the evolutionary tree (110.095; Table 4), while PD was greatest (8880). Ledong is notable among cities in Hainan for having the least economic development, compared to Haikou, which has among the most [43]. Therefore, unsurprisingly, measures of human activity in Ledong, such as consumerism [44], are much lower than for Haikou and than for most other administrative units. The relatively lower level of economic development in Ledong and corresponding human activities may have facilitated maintenance of a high level of endemic diversity, including of rare, recently-emerged species. Together these mechanisms could explain the high PD and low MNTD. This may also be enhanced by the low population density in Ledong [45], which is a county-type administrative unit, and long-term cultivation and management of native medicinal plants by the indigenous Li people [46]. Nevertheless, Ledong is unlikely to be sheltered from the effects of developmental activities for long, there are a lot of endemic plant species harbored in this island, e.g., *Rhamnus hainanensi* Merr. et Chun (Rhamnaceae), *Chunia bucklandioides* H. T. Chang (Hamamelidaceae) and *Madhuca hainanensis* Chun et How (Fig 5). For example, just beyond the Jianfengling National Nature Reserve of Ledong County there is a farm where red dragon fruits [*Hylocereus undatus* ’Foo-Lon’ (Cactaceae); of New World origin] are cultivated. Formerly, this area comprised secondary forests which have been entirely removed for this expansive agricultural activity. Ultimately, the expansion of commercial agriculture in Ledong and other southeastern areas of Hainan will almost assuredly lead to habitat loss for endemic species [47].

Based on an analysis of Jenk’s breaks, we discovered that the number of endemic species in Hainan’s northeastern administrative units was significantly lower than the southwestern units (Fig 2). This may be because the northeastern part of Hainan has fewer mountains than the southwest [48], and mountains are known to have a positive relationship to the number of species within a given area [49–51], though the relationship between elevation to species endemism is less well known [52].

In this study, we found that the plant taxonomic and phylogenetic diversity were significantly correlated with industrial and agricultural revenue and population in Hainan, while natural environmental variables were not significantly correlated with phylogenetic diversity indexes (Table 5). In other words, anthropogenic variables play the dominant role in shaping the endemic plant taxonomic and phylogenetic diversity on Hainan. While this result is jarring, it remains unclear at what point in the history of Hainan anthropogenic factors overtook natural environmental ones as the primary drivers of the organization of botanical diversity on the island. Notably, our detection of a weak but significant latitudinal diversity gradient of endemic species may suggest the historical signature of the roles of natural factors, as latitudinal diversity gradient is a well-known, natural pattern in the organization of biodiversity [53–55]. Broadly speaking, the dominant role of anthropogenic variables likely arises from the rapid social and economic development of Hainan in the past decades. One major type of recent development in has been tourism, which is now one of the island’s main industries and its initial rise to economic importance was also accompanied by ecological destruction. Thus, the development of the tourism industry may have been the/a major catalyst in the shift from natural to anthropogenic drivers of diversity. However, these and other non-mutually exclusive alternatives remain to be tested, such as by utilizing historical specimen records and species lists.

Hainan is one of the richest provinces of China in terms of botanical biodiversity [8, 9, 56]. Compared to the other provinces of China globally well-known for their biodiversity (e.g., Yunnan and Guangxi), the number of native plant species per sq km is higher in Hainan [11]. Therefore, Hainan merits considerable attention for continued national conservation efforts. From a botanical perspective, the distribution of species and patterns of diversity depend on both long-term evolutionary processes and current environmental interactions of individual organisms. Thus, one approach to conservation is to identify hotspots containing plants that have, overall, limited geographic distributions [57]. Such species include endemic species of Hainan, which are limited to the island or some subregion on it. Thus, our study, using PD and focusing on endemic species provides a preliminary guide for identifying sites with potential high conservation need and value, such as in Ledong. Moreover, we believe that the summary of phenology of endemic species that we provide here will be valuable for assessing future phenological shifts, which are associated with vegetation turnover and climate change [58].

## Supporting information

S1 Appendix Variables used in this study to infer the drivers of plant diversity and phylogenetic diversity in Hainan. (DOC)

S2 Appendix Phylogenetic tree of 387 endemic plant species of Hainan based on Phylomatic of the Phylocom software to assemble a phylogenetic tree to estimate branch lengths and calculate phylogenetic metrics. (XLS)

S3 Appendix R script to obtain the goodness of fit for two to 18 breaks with the function plotJenks of the GmAMisc library v. 1.1.1 (https://github.com/gianmarcoalberti/GmAMisc). (R)

S4 Appendix Based on the goodness of fit scores, we selected the least breaks beyond which adding more breaks yielded considerably less gains (i.e., elbow method). (PDF)
