# Correction: Anthropogenic factors are stronger drivers of patterns of endemic plant diversity on Hainan Island of China than natural environmental factors

**DOI:** 10.1371/journal.pone.0274986

**Published:** 2022-09-15

**Authors:** The PLOS ONE Staff

The third author’s name is spelled incorrectly. The correct name is: AJ Harris. The correct citation is:

Zhu ZX, Nizamani MM, Harris A, Wang HF (2021) Anthropogenic factors are stronger drivers of patterns of endemic plant diversity on Hainan Island of China than natural environmental factors. PLOS ONE 16(9): e0257575. https://doi.org/10.1371/journal.pone.0257575

The publisher apologizes for the error.
