# Supplementary material for: Anthropogenic factors are stronger drivers of patterns of endemic plant diversity on Hainan Island of China than natural environmental factors
Source: PLoS One. 2021 Sep 29;16(9):e0257575. doi: 10.1371/journal.pone.0257575 (PMC8480898; doi:10.1371/journal.pone.0257575)
Supplement: S1 Appendix — (DOC) [file pone.0257575.s001.doc]

**Anthropogenic Factors are Stronger Drivers of Patterns of Endemic Plant Diversity on Hainan Island of China than Natural Environmental Factors**

Zhi-Xin Zhu^1^, Mir Muhammad Nizamani^1^, AJ Harris^2^, Hua-Feng Wang^1*^

^1^Hainan Key Laboratory for Sustainable Utilization of Tropical Bioresources, College of Tropical Crops, Hainan University, Haikou, China

^2^Key Laboratory of Plant Resources Conservation and Sustainable Utilization, South China Botanical Garden, Chinese Academy of Science, Guangzhou 510650, China

Appendix 1 Variables used in this study to infer the force driving plant diversity and phylogenetic diversity in Hainan.

|  | Population in 2013 | Agriculture Revenue(10 thousand Yuan) in 2013 | Industry Revenue(10 thousand Yuan) in 2013 | The number of Tourists in 2013 | Area (km^2^) | Min Temperature of Coldest Month (BIO6) | Max Temperature of Warmest Month (BIO5) | Annual Precipitation (BIO12)  (mm) | Mean Diurnal Range (BIO2) |
| --- | --- | --- | --- | --- | --- | --- | --- | --- | --- |
| Baisha | 194667 | 96038 | 24546 | 278183 | 2117.73 | 18 | 32 | 1725 | 2202 |
| Baoting | 172374 | 115393 | 5648 | 360786 | 1166.6 | 17 | 29 | 2050 | 2201 |
| Changjiang | 263823 | 156569 | 441445 | 278183 | 1569 | 19 | 33 | 1676 | 2100 |
| Chengmai | 570233 | 378273 | 729745 | 661435 | 2072 | 18 | 32 | 1786.1 | 2059 |
| Danzhou | 987406 | 362443 | 116261 | 1185767 | 3394 | 3.2 | 27.8 | 1815 | 1880 |
| Ding'an | 341102 | 201511 | 33044 | 573602 | 1189 | 17 | 31 | 1953 | 2198 |
| Dongfang | 458595 | 334979 | 483937 | 517286 | 2266.62 | 1.4 | 38.8 | 1150 | 2100 |
| Haikou | 1632328 | 373518 | 1338134 | 10443089 | 2,237 | 2.8 | 39.6 | 1,650 | 2070 |
| Ledong | 545388 | 512395 | 21525 | 331779 | 2747 | 16 | 32 | 1 559.3 | 2269 |
| Lin'gao | 512217 | 182455 | 47162 | 274880 | 1317 | 16.9 | 28.3 | 1417.8 | 2200 |
| Lingshui | 386432 | 191738 | 14196 | 1391250 | 1128 | 20 | 30 | 2000 | 2100 |
| Qionghai | 506582 | 516184 | 71854 | 2081099 | 1,692 | 15.8 | 33 | 2,060 | 2037 |
| Qiongzhong | 233888 | 102994 | 10620 | 289800 | 2704.66 | 16 | 38 | 1800 | 2322 |
| Sanya | 576927 | 458997 | 171058 | 12283977 | 1,919.58 | 5.1 | 35.9 | 1,392.10 | 2334 |
| Tunchang | 313517 | 153074 | 10815 | 294472 | 1231.5 | 16 | 29 | 2180 | 2000 |
| Wanning | 633897 | 307896 | 45397 | 3321367 | 4443.6 | 18.7 | 28.5 | 2400 | 2000 |
| Wenchang | 596831 | 377108 | 87092 | 1372591 | 2,403 | 6.6 | 36 | 1721.6 | 1953.8 |
| Wuzhishan | 112607 | 32469 | 7847 | 525628 | 1169 | 17 | 35.9 | 2810.4 | 1800 |
