# Supplementary figures and images for: Anthropogenic factors are stronger drivers of patterns of endemic plant diversity on Hainan Island of China than natural environmental factors
Source: PLoS One. 2021 Sep 29;16(9):e0257575. doi: 10.1371/journal.pone.0257575 (PMC8480898; doi:10.1371/journal.pone.0257575)

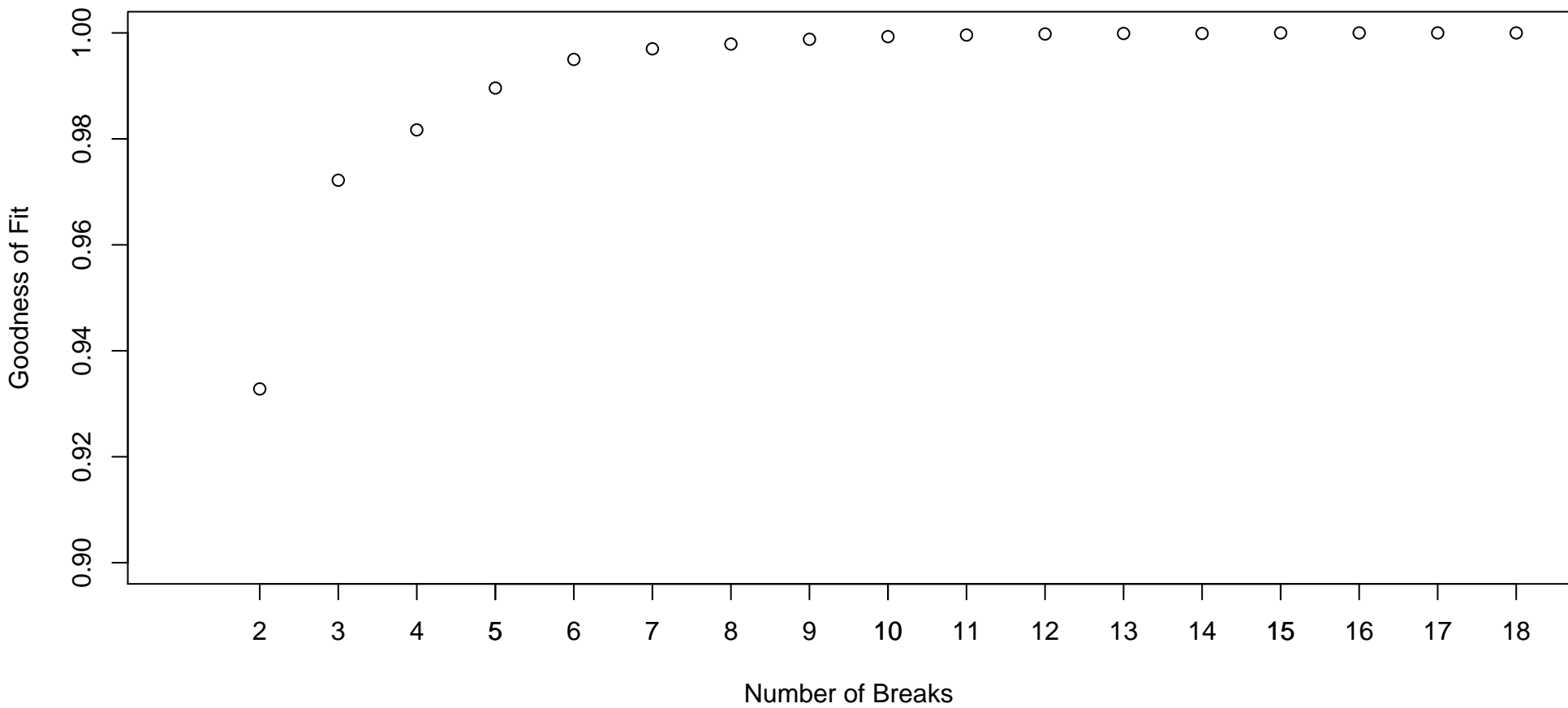

Supplement: S4 Appendix — (PDF) [file pone.0257575.s004.pdf]
